# On the Pernicious Effects of Colchicum, and Other Gout Medecines

**Published:** 1822-06

**Authors:** W. Norman


### THERAPEIA.

### ART. IV.—*On the pernicious effects of Colchicum, and other Gout Medecines.* By W. NORMAN, Esq.

AT a moment like the present, when the public mind is vacillating, in "fearful suspence," between the conflicting opinions of medical practitioners on the real properties of colchicum autumnale, and the questionable propriety of employing it as a remedy for gout, it is incumbent on every one who has witnessed the effects of that medicine on the human constitution to step forward, and publicly propound the results of his observations,—notwithstanding they should be at variance with the dicta of higher authorities, or in opposition to deductions drawn from the extended walks of metropolitan practice,—in order that this momentous question may be at once definitively settled on legitimate and unerring grounds. Nor, indeed, (though it be too frequently the case,) should any writer be deterred from so laudable an undertaking, because he is unable to dress out his facts in the imitative garb of scientific illustration; or through fear of the conceited denunciations of *a* critical "WE;" for a "plain unvarnished tale" of practical truths contributes infinitely more to the relief of human sufferings, and the advancement of useful science, than volumes of far-fetched, speculative theories, rendered unintelligible by the pompous obscurities of pedantry and affectation.

Early allured by the seductive character in which colchicum autumnale was introduced to public notice, and speedily conceding to the irresistible authorities that graced and dignified its *entrè* into medical practice; yet not altogether forgetful of the excellent maxim "*fronti nulla fides*;" I resolved to submit its "*vis medica*" (as the learned have it,) to the conclusive tests of experiment, confident that my expectations in its remedial powers would be amply realized in its remedial products. So, indeed, for a season, they were. Gouty patients from time to time required my assistance: colchicum autumnale was successively, but cautiously, employed, first in the form of tincture, and subsequently in every "vast variety of form" that presented itself; till, at length, I concluded that a safe and certain remedy was at last possessed for the cure of a malady which had hitherto baffled human skill, and which had justly received the denomination of "*crux medicorum*" on that account.

I had not long, however, slumbered on this "bed of roses," before my delusive reveries were broken by the intelligence of the death of a relation, within twenty-four hours after having taken half a bottle of eau medicinale, under circumstances that could leave no doubt whatever that his death was occasioned by the use of that remedy. Time, likewise, brought me further intelligence on the same subject; and I soon had the mortification to learn that those patients whom I had several times speedily rescued from the agonizing tortures of gout, through the medium of guarded doses of colchicum autumnale, were now enduring relapses of disease, after much shorter enjoyment of much less satisfactory intervals of health than usual. Simultaneously with these events, indubitable authorities apprised me, also, of many other unpropitious sequelæ to the well-directed employment of the medicine in question; and a summons to attend at a consultation in a case of apoplexy, supervening to a taken dose of "Reynolds' specific," finally determined me to thenceforth abandon its adoption for ever.

Thus, then, from observation and experience of no inconsiderable extent, my mind is fully persuaded that colchicum autumnale is not, under any modification of form whatever, which I am at present acquainted with, a safe and efficient remedy for gout; and that he who confides in its prophylactic influence on that disease will, sooner or later, be woefully disappointed by its opposite consequences.

*Langport; April 12th*, 1822.

